# Application of Step-by-Step and Paediatric Emergency Care Applied Research Network (PECARN) Clinical Decision Aids in the management of young febrile infants in a UK cohort

**DOI:** 10.1136/emermed-2025-214876

**Published:** 2025-10-06

**Authors:** Etimbuk Umana, Hannah Norman-Bruce, Clare Mills, Oenone Rodgers, Hannah Mitchell, Lisa McFetridge, Gareth McKeeman, Steve Foster, Michael Barrett, Damian Roland, Mark D Lyttle, Chris Watson, Thomas Waterfield, on behalf of Paediatric Emergency Research in the UK and Ireland (PERUKI), Phillipa Rawling

**Affiliations:** 1 Wellcome-Wolfson Institute for Experimental Medicine, Queen’s University Belfast, Belfast, Northern Ireland, UK; 2 Queen’s University Belfast School of Mathematics and Physics, Belfast, Northern Ireland, UK; 3 Cinical Chemistry, Belfast Health and Social Care Trust, Belfast, UK; 4 Royal Hospital for Children Glasgow, Glasgow, UK; 5 Emergency Medicine, Children’s Health Ireland at Crumlin, Dublin, Ireland; 6 Women’s and Children’s Health, University College Dublin, Dublin, Ireland; 7 Paediatric Emergency Medicine Leicester Academic (PEMLA) Group, Children’s Emergency Department, Leicester Royal Infirmary, Leicester, Leicester, UK; 8 Health Sciences, University of Leicester, Leicester, UK; 9 Emergency Department, Bristol Royal Children’s Hospital, Bristol, UK; 10 Emergency Department, Royal Belfast Hospital for Sick Children, Belfast, UK

**Keywords:** bacterial, infections, Diagnostic Tests

## Abstract

**Background:**

Young febrile infants are at high risk of invasive bacterial infections (IBIs). Clinical Decision Aids (CDA) such as the Step-by-Step and Paediatric Emergency Care Applied Research Network (PECARN) use Procalcitonin (PCT), limiting their application in settings without PCT access. This study aimed to test the performance of these CDAs in a UK cohort.

**Methods:**

This was a planned analysis of the Febrile Infant Diagnostic Assessment and Outcome Study, a large, prospective multicentre observational study conducted across over 30 sites in the UK. Febrile infants (0–90 days of age) with complete biomarker data, who also underwent PCT testing, were included. Two CDAs, PECARN and Step-by-Step, were applied to the cohort, using their recommended low-risk criteria. The diagnostic performance of the CDAs was analysed.

**Results:**

Of the 1527 infants who completed biomarker testing in the main study, 442 had PCT testing and were included, 22 (5%) were diagnosed with an IBI. PECARN and Step-by-Step CDAs demonstrated sensitivities of 1.00 (95% CI: 0.85 to 1.00) and 0.96 (95% CI: 0.77 to 1.00) respectively. The PECARN CDA performed with a specificity of 0.14 (95% CI: 0.11 to 0.18) identifying 14% of the participants as low-risk and did not misclassify any infants. The Step-by-Step CDA performed with a specificity of 0.15 (95% CI: 0.12 to 0.19) identifying 14% of the participants as low-risk and misclassifying one participant with IBI as low-risk.

**Conclusion:**

Both PECARN and Step-by-Step CDAs demonstrated high sensitivity for detecting IBI in our cohort. While specificity was relatively low, these tools could potentially identify a subset of low-risk infants suitable for less intensive management.

**WHAT IS ALREADY KNOWN ON THIS TOPIC**

**WHAT THIS STUDY ADDS**

- Both CDAs are dependent on Procalcitonin (PCT) which is not routinely available in the UK. This study provides the first application of the Step-by-Step and PECARN CDAs to the UK population who had PCT testing.

**HOW THIS STUDY MIGHT AFFECT RESEARCH, PRACTICE OR POLICY**

- The PECARN and Step-by-Step CDAs identified a low-risk cohort of infants suitable for management without parenteral antibiotics and lumbar puncture. The reported performance was similar to other CDAs, including those not dependent on PCT.

## Introduction

 Febrile infants under 91 days of age are at a relatively high risk of invasive bacterial infections (IBIs), namely bacterial meningitis and bacteraemia.^1^,^3^ The international prevalence of IBI among febrile infants ranges from 1% to 4% and if undiagnosed, is associated with high morbidity and mortality.^24^,^8^ To this end, infants have traditionally undergone thorough assessment with blood and urine testing, the majority receiving parenteral antibiotics and undergoing lumbar puncture.^3 5 8^

However, the management of febrile infants has seen a paradigm shift over the last two decades with validated Clinical Decision Aids (CDA) developed in North America and Europe. These CDAs aim to safely identify a low-risk cohort of febrile infants who are suitable for management without invasive tests, parenteral antibiotics and hospital admission.^2^,^49^ The benefits of identifying a low-risk cohort are extensive, from safely allocating limited healthcare resources to avoiding long-term harms associated with early antibiotic exposures.^3 10^ These validated, and widely used, CDAs typically determine risk using sequential assessment based on the infant’s age (those under 21 or 28 days deemed higher risk) urinalysis results, clinical appearance and blood biomarkers.^2 3 11^

The current authors recently undertook the Febrile Infant Diagnostic Assessment and Outcome (FIDO) study, looking at CDAs in febrile infants in the UK and Ireland.^8^ C-reactive protein (CRP) based CDAs such as the British Society for Antimicrobial Chemotherapy (BSAC) and the American Academy of Pediatrics (AAP) reported sensitivity of 0.99 (95% CI: 0.92 to 1.00). The reported specificity for both BSAC and AAP was above 0.20. A subset of infants in the FIDO study had Procalcitonin (PCT) available allowing comparison of the AAP CDA using CRP or PCT. There was no difference in performance between both CDAs (p=1.000). However, in our cost analysis, the AAP CDA using PCT was more costly than AAP CDA using CRP (£1396 vs £1278 per infant).^8^ The Paediatric Emergency Care Applied Research Network (PECARN) and the Step-by-Step CDAs have been validated in North America and Europe, but no study has evaluated their use in a UK cohort. One of the main limitations is that these CDAs use PCT, which is not readily available in the UK, making their application to the UK challenging. Though CRP is readily available in the UK, evidence from a systematic review and meta-analysis reported better diagnostic accuracy for PCT (0.5 ng/L) compared with CRP (20 mg/mL) for IBI.^12^

The aim of this planned, sub-analysis of the FIDO study cohort is to apply the PECARN and Step-by-Step CDAs in a UK cohort with PCT available for detection of IBI.

## Methods

The FIDO study was a prospective observational cohort study conducted across the Paediatric Emergency Research in the UK and Ireland (PERUKI) network. The protocol and methodology have been previously published.^13^ This study has been reported in line with STROBE (Strengthening the Reporting of OBservational studies in Epidemiology) and STARD (Standards for Reporting Diagnostic Accuracy) criteria for reporting diagnostic test accuracy studies guidance.^14 15^

### Setting

30 PERUKI sites prospectively recruited febrile infants and collected samples for PCT analysis. The study sites included 1 in Northern Ireland, 1 in Wales, 2 in Scotland and 26 in England, with recruitment taking place from July 2022 to August 2023. The details and recruitment rates of sites that participated in PCT analysis can be seen in table 1.

**Table 1:** Site characteristics and recruitment.

Site code	Site population	N=442 n (%)
Site 1	Mixed (adults and paediatric)	9 (2.0)
Site 2	Mixed (adults and paediatric)	4 (0.9)
Site 3	Paediatric only	37 (8.4)
Site 4	Mixed (adults and paediatric)	6 (1.4)
Site 5	Mixed (adults and paediatric)	10 (2.3)
Site 6	Paediatric only	26 (5.9)
Site 7	Paediatric only	13 (2.9)
Site 8	Mixed (adults and paediatric)	14 (3.2)
Site 9	Paediatric only	5 (1.1)
Site 10	Mixed (adults and paediatric)	14 (3.2)
Site 11	Paediatric only	4 (0.9)
Site 12	Mixed (adults and paediatric)	21 (4.8)
Site 13	Paediatric only	21 (4.8)
Site 14	Paediatric only	19 (4.3)
Site 15	Paediatric only	31 (7.0)
Site 16	Paediatric only	13 (2.9)
Site 17	Paediatric only	9 (2.0)
Site 18	Paediatric only	19 (4.3)
Site 19	Paediatric only	20 (4.5)
Site 20	Paediatric only	9 (2.0)
Site 21	Paediatric only	41 (9.3)
Site 22	Paediatric only	21 (4.8)
Site 23	Mixed (adults and paediatric)	4 (0.9)
Site 24	Mixed (adults and paediatric)	17 (3.8)
Site 25	Mixed (adults and paediatric)	17 (3.8)
Site 26	Paediatric only	15 (3.4)
Site 27	Paediatric only	7 (1.6)
Site 28	Paediatric only	8 (1.8)
Site 29	Mixed (adults and paediatric)	2 (0.5)
Site 30	Mixed (adults and paediatric)	6 (1.4)

### Participants

Infants ≤90 days presenting with a fever of ≥38°C recorded using any thermometer in the emergency department (ED)/assessment unit (AU) or by anyone within the preceding 24 hours of presentation and had PCT available for analysis were eligible for inclusion in this substudy of the FIDO cohort. Infants without consent from parents or guardians, those without PCT available for analysis and infants with incomplete data or biomarkers (absolute neutrophil count (ANC) and CRP) were excluded.

### Study procedures

Study procedures have been previously reported.^8 13^ Briefly, infants presenting to the ED or AU were screened for eligibility by clinical staff on arrival to the hospital. These infants were cared for according to local practice and managed without delay. During the initial evaluation, clinicians completed a contemporaneous case report form (CRF), documenting the initial assessment and clinical findings prior to consent discussions. During routine phlebotomy on presentation to ED/AU, an additional 1 mL of blood was collected in an EDTA blood tube for PCT analysis, where feasible. Where sample volumes were short, standard care was prioritised. When obtained, samples were sent to the local laboratory to be processed and stored as plasma at −80°C for PCT analysis. Research without prior consent methodology (RWPC) was used as described in the FIDO protocol.^13^ Parents or guardians of infants were consented once the clinical condition of the infant was stable and as directed by the clinical team. RWPC has been used in multiple multicentre studies in emergency care where clinical care must be delivered without delay.^16^,^19^ No blood samples were transferred out of the participating centres without consent being obtained. At the end of the study, samples were transferred to Queen’s University Belfast and PCT analysis was undertaken at the Department of Clinical Biochemistry Laboratory, Royal Victoria Hospital Belfast. All samples underwent PCT analysis using the Elecsys BRAHMS PCT assay (Thermo Fisher Scientific) on the Roche e801. Staff conducting the PCT analysis were blinded to all study clinical data and reference standards. The CRP and ANC testing was performed at local accredited National Health Service (NHS) laboratories as part of the infant’s routine investigations. A 7-day postdischarge CRF was completed by the study team which included results of investigations, disposition from ED/AU, antibiotic administration, paediatric intensive care outcomes, ED/AU length of stay, ward length of stay and reattendance within 7 days.

### Reference standards

IBI was defined as the identification of a bacterial pathogen in either the blood or cerebrospinal fluid (CSF) via culture or molecular testing methods in laboratories accredited by the UK Accreditation Service and the NHS. The reference standard test was undertaken by personnel blinded to the clinical data and the presumptive diagnosis. In instances where not all infants underwent blood/CSF cultures or molecular testing, follow-up was conducted via chart reviews 7 days postdischarge to identify any manifestation of IBI.

### Data management and study definitions

Research Electronic Data Capture (REDCap) is a secure, web-based software platform designed to support data capture for research studies.^20^ CRFs were uploaded onto REDCap and managed accordingly. The PECARN and Step-by-Step CDAs were applied using their low-risk criteria as shown in online supplemental table 1.

Urinalysis criteria: (1) abnormal urinary dipstick test (leucocyte esterase ≥1+, or nitrite ≥trace) or (2) abnormal urine microscopy (≥10 white cells per high-power field in centrifuged urine) (3) where urinalysis was not obtained and no urinary tract infection (UTI) within 7-day follow-up postdischarge, was considered to be normal. An infant was considered unwell if they had an abnormal global assessment as reported by the treating clinician, and/or abnormal vital signs (heart rate, respiratory rate and capillary refill time, based on advanced paediatric life support (UK) recommendation for age) on initial assessment.^8 21^

### Statistical analysis

As this was a pragmatic observational sub-study within the FIDO cohort, no formal power calculation was undertaken. The sample comprised all eligible infants with PCT, ANC and CRP during the study period, totalling 442 participants.

Descriptive analysis was undertaken for baseline data. Data were reported as medians with IQRs for continuous variables and proportions for categorical variables. Differences were compared with the Mann-Whitney U test and the χ² or Fisher’s exact test for continuous and categorical variables respectively. The characteristics and outcomes of infants were compared between those who underwent PCT testing and those who did not, as well as between those with IBI and those without IBI.

Diagnostic performance in terms of sensitivity, specificity, positive predictive value, negative predictive value (NPV), positive likelihood ratio and negative likelihood ratio was reported for diagnosis of IBI. An α critical value of 0.05 was regarded as statistically significant. However, a Bonferroni correction was undertaken to limit the impact of multiple testing (α/n), so that our target p value was 0.002. Statistical analyses were performed using MedCalc V.22.021 software (MedCalc Software, Ostend, Belgium).

## Results

**Figure 1:**
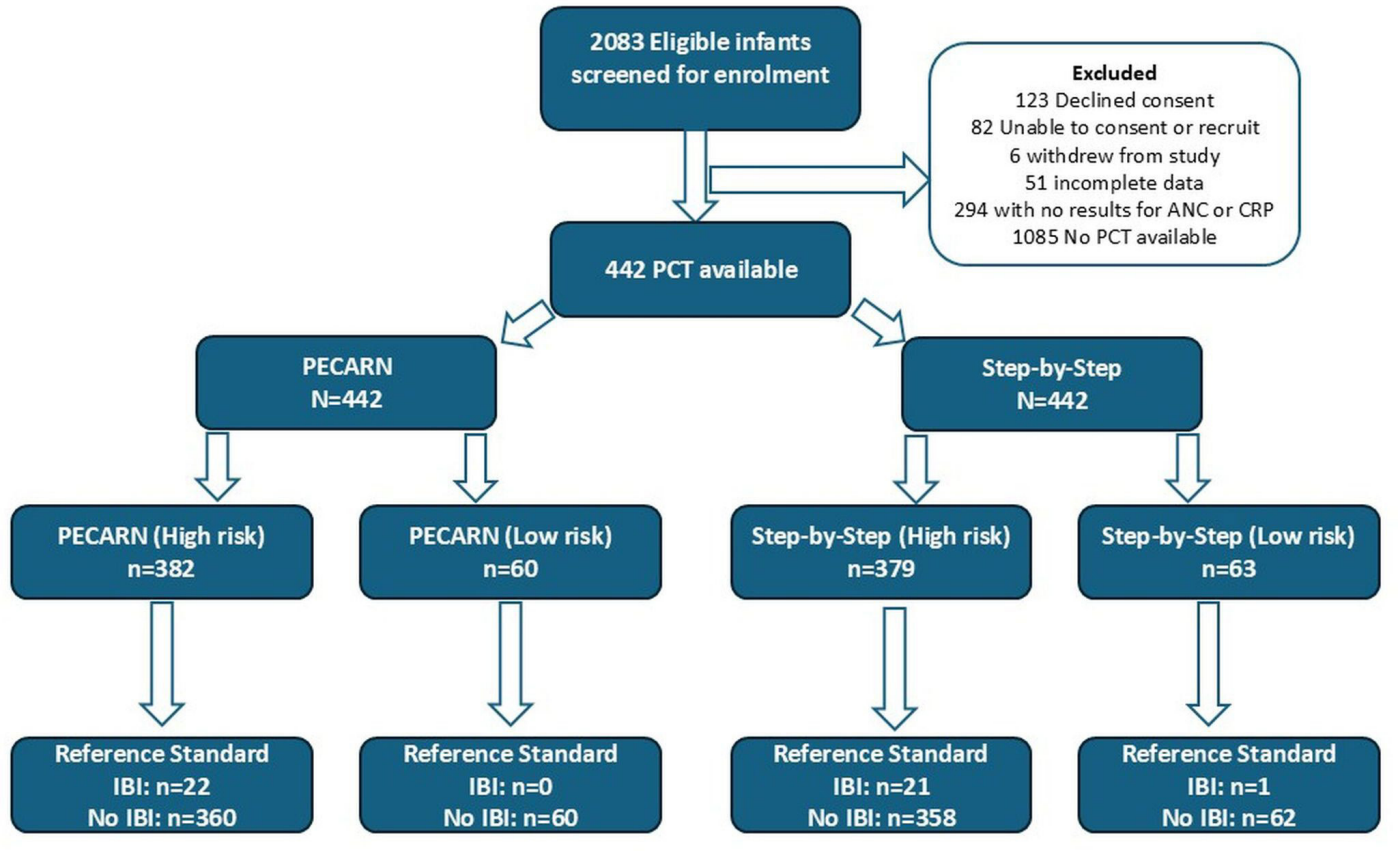
Flow of participants included in the analysis with Clinical Decision Aids and invasive bacterial infection outcomes. ANC, absolute neutrophil count; CRP, C-reactive protein; IBI, invasive bacterial infection; PCT, Procalcitonin; PECARN, Paediatric Emergency Care Applied Research Network.

During the FIDO study period, 1821 infants were enrolled, 1527 underwent biomarker testing and 442 had PCT results available (figure 1). Of the 30 participating sites, 12 were mixed (adult and paediatric) departments, while 18 were paediatric only. The median recruitment per site for infants with PCT was 13.5 (IQR: 7–20) (table 1). The demographic and clinical characteristics of those participants with PCT testing and those without were similar (online supplemental table 2).

The median age of the study cohort was 48 days (IQR 30–66) with 68 (15%) being less than 21 days of age. There were 274 (62%) males, 289 (65%) infants reported to be unwell appearing at presentation to hospital. Blood cultures and/or molecular diagnostic tests were conducted in 417 infants (94%), while CSF cultures and/or molecular methods for pathogen identification were performed in 263 infants (60%). Blood or CSF culture/molecular tests were not performed on 24 infants; they were classified as not having IBI according to a 7-day follow-up reference standard. Of the 442 infants, 22 (5%) were diagnosed with IBI. Infants with IBI were more likely to have longer hospital stays (table 2). Of the 22 with IBI, all had bacteraemia and 1 had both bacteraemia and bacterial meningitis, Streptococcus agalactiae (Group B Streptococcus (GBS). *E. coli* was the most common IBI (11 (50%) (online supplemental table 3). A total of 3 (0.7%) infants from our study cohort were admitted to the paediatric intensive care unit.

**Table 2:** Patient investigations and outcomes.

Variable	Full cohort with PCT testing N=442	IBI n=22	No IBI n=420	P value
Patient characteristics				
Age (days) Median (IQR)	48 (30–66)	42 (32–63)	48 (30–67)	0.757
Sex (male), n (%)	274 (62)	17 (77.3)	257 (61.2)	0.130
Unwell appearing, n (%)	289 (65)	18 (81.8)	271 (64.5)	0.111
Temperature on arrival (C), median (IQR)	38.0 (37.4–38.4)	37.9 (37.0–38.3)	38.0 (37.4–48.4)	0.399
Presenting ≤6 hours from fever onset, n (%)	232 (53)	11 (50)	221 (53)	0.694
Fever with source, n (%)	247 (56)	8 (36)	239 (57)	0.059
Investigations, n (%)				
Respiratory viral test, n (%)	359 (81.2)	14 (63.6)	345 (82.1)	0.030
Urine testing, n (%)	396 (89.6)	20 (90.9)	376 (89.5)	1.000
CSF culture and/or molecular testing, n (%)	263 (59.5)	17 (77.3)	246 (58.6)	0.082
Antibiotics administered, n (%)	338 (76.5)	21 (95.5)	317 (75.5)	0.036
Dispositions, n (%)				0.143
Discharged from ED	28 (6.3)	0 (0)	28 (6.7)	
Ambulated	52 (11.8)	5 (22.7)	47 (11.2)	
Admitted	362 (81.9)	17 (77.3)	345 (82.1)	
ED length of stay, hours (median (IQR))	4 (2–6)	4 (2–6)	4 (2–5.7)	0.968
Ward length of stay, days (median (IQR))	2 (2–4)	5 (2–6.3)	2 (2–3)	<0.001
Unplanned reattendance, n (%)	24 (5.4)	0 (0)	24 (5.7)	0.622

Urine testing included urinalysis, microscopy and culture.

CSF, cerebrospinal fluid; ED, emergency department; IBI, invasive bacterial infection; PCT, Procalcitonin.

### CDA performance CDAs

PECARN and Step-by-Step CDAs demonstrated a sensitivity of 1.00 (95% CI: 0.85 to 1.00) and 0.96 (95% CI: 0.77 to 1.00), respectively, for the exclusion of IBI (table 3). The PECARN CDA performed with a specificity of 0.14 (95% CI: 0.11 to 0.18) identifying 14% of the participants as low-risk and did not misclassify any infants. The Step-by-Step CDA performed with a specificity of 0.15 (95%CI: 0.12 to 0.19) identifying 14% of the participants as low-risk and misclassifying one participant with IBI as low-risk. This infant presented with fever within 6 hours of fever onset (online supplemental table 4).

**Table 3:** Diagnostic performance of PECARN and Step-by-Step CDA.

CDA	Low-risk n (%)	IBI in low risk n (%)	Sensitivity (95% CI)	Specificity (95% CI)	PPV (95% CI)	NPV (95% CI)	PLR (95% CI)	NLR (95% CI)
PECARN	60 (14)	0 (0)	1.00 (0.85 to 1.00)	0.14 (0.11 to 0.18)	0.06 (0.05 to 0.06)	1.0 (0.94 to 1.00)	1.17 (1.12 to 1.21)	0 (0)
Step-by-Step	63 (14)	1 (1.6)	0.96 (0.77 to 1.00)	0.15 (0.12 to 0.19)	0.06 (0.05 to 0.06)	0.98 (0.90 to 1.00)	1.12 (1.01 to 1.24)	0.31 (0.04 to 2.12)

CDA, Clinical Decision Aids; IBI, Invasive bacterial infection; NLR, negative likelihood ratio; NPV, negative predictive value; PECARN, Paediatric Emergency Care Applied Research Network; PLR, positive likelihood ratio; PPV, positive predictive value.

## Discussion

This is the first study to apply the PECARN and Step-by-Step CDAs to a UK multicentre cohort of febrile infants with PCT available. This planned secondary analysis showed both CDAs had good diagnostic performance. The cohort with PCT available had an IBI rate of 5%. Infants with IBI had prolonged hospital admission. Although *E. coli* was the most prevalent bacteria, which aligns with other published literature,^2 4 8^ evidence indicates that different bacteria may initiate distinct immune and biomarker responses.^22^ Therefore, results could vary depending on the prevalence of specific bacteria in different geographical regions.

The PECARN CDA did not misclassify any infants with IBI. It had a sensitivity and NPV of 1.0. The original PECARN study reported sensitivity and NPV of 0.97 and 1.0, respectively, for IBI.^4^ Validation in Spanish and Singaporean cohorts showed sensitivities less than 0.9 and an NPV of 0.9.^23 24^ These findings highlight the challenge of reporting consistent accuracy results for CDAs developed in different jurisdictions. Interestingly, our study reports the lowest specificity of 0.14 compared with other studies which report specificity above 0.30.^23 24^

The Step-by-Step CDA had a reported sensitivity and NPV of 0.96 and 0.98. This sensitivity is comparable to the original validation study.^2^ Other studies have also reported similar diagnostic performance of the Step-by-Step CDA.^24 25^ However, in this UK study, the Step-by-Step CDA misclassified one infant, who presented less than 6 hours from fever onset, with normal urinalysis and biomarkers. This infant had Staphylococcus aureus bacteraemia. With 53% of infants presenting with fever onset of less than 6 hours, caution should be applied when using CDAs in this cohort. These infants may warrant a period of observation or a lower biomarker threshold.^26^

The performance of the PECARN and Step-by-Step CDA is similar to other CDAs applied within the parent FIDO study such as the BSAC and AAP CDA.^8^ When applied to a cohort of febrile infants, the AAP and BSAC CDA could identify more than 20% of infants who are deemed to be low-risk compared with 14% identified as low-risk by the PECARN and Step-by-Step CDAs. Lumbar punctures and antibiotics could be avoided in this group of low-risk infants. All these CDAs use sequential assessment with the main difference being the biomarker of choice, PECARN and Step-by-Step using PCT, while BSAC and AAP use CRP. The parent FIDO study identified a cost difference favouring CRP over PCT when focusing on the AAP CDA, which allows substitution of PCT for CRP.^8 12^

The differences in the performance of the PECARN and Step-by-Step CDA in our cohort can be explained by a number of factors and variation in practice. Specifically, among the study cohort, 65% of participants were deemed to be unwell appearing. This is much higher than the 13% reported in the original Step-by-Step validation study.^2^ Another source of variation could be attributed to differences in population from which these CDAs were derived, with some including infants presenting with a fever with a source (FWS) or exclusively well-appearing infants.^2 4^ Also, there is wide variation in practice between the UK, America and mainland Europe in terms of their clinical assessment of the febrile infants.^2 4 5^ Consequently, the authors opted for the term ‘application’ instead of ‘validation’ to facilitate the adaptation of these CDAs for implementation within the UK’s current practices and population.

This study has several advantages and limitations. This was a prospective study conducted across multiple academic and urban centres with a good geographical spread. Unlike the validation studies for PECARN and Step-by-Step, this study used broader inclusion criteria, including infants with FWS.^2 4^ It is particularly reassuring that the CDAs showed comparable sensitivities to their validation cohorts, despite differences in population and IBI rates. The number of IBI cases was low in our study and could result in a type 2 error. However, the number of IBI in our study is similar in proportion to other published studies of this nature.^24 27^ While the PECARN CDA was originally validated in a cohort of febrile infants less than 60 days of age, evaluation practices in the UK and Ireland extend to infants up to 90 days old, supported by evidence of IBI risk in infants aged 61–90 days.^28 29^ Though this study represents the largest cohort of febrile infants with PCT testing in the UK and Ireland, the wide CIs warrant cautious interpretation. Additionally, potential selection bias exists given the higher proportion of unwell appearing infants and a large proportion of infants did not undergo PCT analysis in the main FIDO cohort.

## Conclusion

This study successfully applied the PECARN and Step-by-Step CDAs to a UK cohort of febrile infants who had PCT testing. These CDAs are capable of identifying a low-risk group where lumbar punctures and antibiotics may be unnecessary. Caution should be exercised for those presenting with a fever onset of less than 6 hours. The study emphasises the challenges of applying CDAs to different contexts with both differing clinical practices and population characteristics.

## Supplementary material

10.1136/emermed-2025-214876 online supplemental file 1

## Data Availability

Data are available upon reasonable request.

## References

[1] Ladhani SN, Henderson KL, Muller-Pebody B (2019). Risk of invasive bacterial infections by week of age in infants: prospective national surveillance, England, 2010-2017. Arch Dis Child.

[2] Gomez B, Mintegi S, Bressan S (2016). Validation of the “Step-by-Step” Approach in the Management of Young Febrile Infants. Pediatrics.

[3] Pantell RH, Roberts KB, Adams WG (2021). Evaluation and Management of Well-Appearing Febrile Infants 8 to 60 Days Old. Pediatrics.

[4] Kuppermann N, Dayan PS, Levine DA (2019). A Clinical Prediction Rule to Identify Febrile Infants 60 Days and Younger at Low Risk for Serious Bacterial Infections. JAMA Pediatr.

[5] Waterfield T, Lyttle MD, Munday C (2022). Validating clinical practice guidelines for the management of febrile infants presenting to the emergency department in the UK and Ireland. Arch Dis Child.

[6] Milcent K, Faesch S, Gras-Le Guen C (2016). Use of Procalcitonin Assays to Predict Serious Bacterial Infection in Young Febrile Infants. JAMA Pediatr.

[7] Curelaru S, Samuel N, Chayen G (2023). Outcomes of Infants Who Are Febrile Aged 29-90 Days Discharged from the Emergency Department. J Pediatr.

[8] Umana E, Mills C, Norman-Bruce H (2024). Performance of clinical decision aids (CDA) for the care of young febrile infants: a multicentre prospective cohort study conducted in the UK and Ireland. EClinicalMedicine.

[9] Umana E, Norman-Bruce H, Waterfield T (2025). Scoping review of clinical decision aids in the assessment and management of febrile infants under 90 days of age. BMC Pediatr.

[10] Schulfer A, Blaser MJ (2015). Risks of Antibiotic Exposures Early in Life on the Developing Microbiome. PLoS Pathog.

[11] National Institute for Health and Care Excellence (2019). Fever in under 5s: assessment and initial management nice guideline ng143. NICE Guidelines Online.

[12] Norman-Bruce H, Umana E, Mills C (2024). Diagnostic test accuracy of procalcitonin and C-reactive protein for predicting invasive and serious bacterial infections in young febrile infants: a systematic review and meta-analysis. *The Lancet Child & Adolescent Health*.

[13] Umana E, Mills C, Norman-Bruce H (2023). Applying clinical decision aids for the assessment and management of febrile infants presenting to emergency care in the UK and Ireland: Febrile Infant Diagnostic Assessment and Outcome (FIDO) Study protocol. BMJ Open.

[14] von Elm E, Altman DG, Egger M (2008). The Strengthening the Reporting of Observational Studies in Epidemiology (STROBE) statement: guidelines for reporting observational studies. J Clin Epidemiol.

[15] Bossuyt PM, Reitsma JB, Bruns DE (2015). STARD 2015: an updated list of essential items for reporting diagnostic accuracy studies. BMJ.

[16] CONNECT advisory group (2015). Research without prior consent (deferred consent) in trials investigating the emergency treatment of critically ill children.

[17] Waterfield T, Lyttle MD, Shields M (2019). Parents’ and clinicians’ views on conducting paediatric diagnostic test accuracy studies without prior informed consent: qualitative insight from the Petechiae in Children study (PiC). Arch Dis Child.

[18] Waterfield T, Maney J-A, Fairley D (2021). Validating clinical practice guidelines for the management of children with non-blanching rashes in the UK (PiC): a prospective, multicentre cohort study. Lancet Infect Dis.

[19] Lyttle MD, Rainford NEA, Gamble C (2019). Levetiracetam versus phenytoin for second-line treatment of paediatric convulsive status epilepticus (EcLiPSE): a multicentre, open-label, randomised trial. The Lancet.

[20] Harris PA, Taylor R, Minor BL (2019). The REDCap consortium: Building an international community of software platform partners. J Biomed Inform.

[21] Smith S (2023). Advanced Paediatric Life Support: A Practical Approach to Emergencies.

[22] Gangoiti I, Fernandez C-L, Gallego M (2021). Markers for invasive bacterial infections in previously healthy children. Am J Emerg Med.

[23] Velasco R, Gomez B, Benito J (2021). Accuracy of PECARN rule for predicting serious bacterial infection in infants with fever without a source. Arch Dis Child.

[24] Sutiman N, Khoo ZX, Ong GYK (2022). Validation and comparison of the PECARN rule, Step-by-Step approach and Lab-score for predicting serious and invasive bacterial infections in young febrile infants. Ann Acad Med Singap.

[25] Yang Y, Wang Y-M, Lin C-HR (2023). Explainable deep learning model to predict invasive bacterial infection in febrile young infants: A retrospective study. Int J Med Inform.

[26] Velasco R, Gomez B, Labiano I (2024). Performance of Febrile Infant Algorithms by Duration of Fever. *Pediatrics*.

[27] Rabiner JE, Capua M, Golfeiz D (2019). Validation of Risk Stratification Criteria to Identify Febrile Neonates at Low Risk of Serious Bacterial Infection. Glob Pediatr Health.

[28] Umana E, Norman-Bruce H, Mills C (2023). Applying the American Academy of Pediatrics guideline to a cohort of febrile infants attending emergency departments in the UK and Ireland. Eur J Emerg Med.

[29] Umana E, Waterfield T (2025). Prevalence of Invasive Bacterial Infection Among Febrile Infants Aged 61 to 90 Days. *JAMA Netw Open*.

