# Supplementary material for: Application of Step-by-Step and Paediatric Emergency Care Applied Research Network (PECARN) Clinical Decision Aids in the management of young febrile infants in a UK cohort
Source: Emerg Med J. 2025 Oct 6;43(3):e214876. doi: 10.1136/emermed-2025-214876 (PMC13018732; doi:10.1136/emermed-2025-214876)
Supplement: online supplemental file 1 [file emermed-43-3-s001.docx]

**EMJ supplements**

**Application of Step-by-Step and Pediatric Emergency Care Applied Research Network (PECARN) Clinical Decision Aids in the management of young febrile infants in a UK cohort.**

Contents

[Table 1: Description of Clinical Decision Aids (CDA)s, their low risk criteria and derivation methods. 1](#_Toc207249450)

[Table 2: Patient characteristics in infants with or without PCT (Total cohort -1527). 2](#_Toc207249451)

[Table 3: Organism identified for IBI 2](#_Toc207249452)

[Table 4: Infants with IBI misclassified by the CDAs Step-by-Step 3](#_Toc207249453)

## Table 1: Description of Clinical Decision Aids (CDA)s, their low risk criteria and derivation methods.

| **CDA** | **Low risk criteria^a^** | **Year of**  **derivation** | **Derivation**  **Methodology** |
| --- | --- | --- | --- |
| PECARN^b^ | Negative urinalysis, ANC <4.0 and PCT <0.5ng/ml | 2019 | Recursive partitioning |
| Step-by-Step | >21 days, well appearing, negative urinalysis,  PCT <0.5, ANC<10.0 or CRP<20mg/l | 2014 | Expert Consensus and  Evidence Synthesis |
| ANC-Absolute Neutrophil count, PCT-Procalcitonin, CRP-C-reactive Protein  (a)- Infants were only classified as low risk if all criteria were present.  (b)- Unwell appearing infants were classified as high risk along with the PECARN diagnostic criteria. | | | |

## Table 2: Patient characteristics in infants with or without PCT (Total cohort -1527).

| **Variable** | **Non PCT group**  **n-1085 (%)** | **PCT group**  **n-442 (%)** | **P value** |
| --- | --- | --- | --- |
| **Age Category** |  |  | 0.082 |
| </= 21 days | 191 (17.6) | 68 (15.4) |  |
| 22 – 28 days | 82 (7.6) | 36 (8.2) |  |
| 29 – 60 days | 521 (48.0) | 192 (43.4) |  |
| 61 – 90 days | 291 (26.8) | 146 (33.0) |  |
| **Gender** |  |  | 0.430 |
| Male | 649 (59.8) | 274 (62.0) |  |
| Female | 436 (40.2) | 168 (38.0) |  |
| **Comorbidities present** | 160 (14.7) | 67 (15.2) | 0.837 |
| **Presenting ≤ 6hours from fever onset** | 589 (54.3) | 232 (52.5) | 0.064 |
| **Fever with source** | 594 (54.7) | 247 (55.9) | 0.686 |
| **Temperature > 38C on arrival** | 600 (55.3) | 239 (54.1) | 0.662 |
| **Unwell appearing** | 643 (59.3) | 289 (65.4) | 0.026 |
| **IBI rate** | 40 (3.7) | 22 (5.0) | 0.246 |
| IBI-Invasive bacterial infection, PCT-Procalcitonin | | | |

Table 3: Organism identified for IBI.

| **Organism IBI** | **n-22 (%)** |
| --- | --- |
| **Escherichia coli*** | 11 (50) |
| **Neisseria meningitidis***** | 2 (9) |
| **Staphylococcus aureus** | 5 (23) |
| **Streptococcus agalactiae (GBS)**** | 3 (14) |
| **Streptococcus gallolyticus** | 1 (4) |
| *1 patient diagnosed with E. coli bacteremia also cultured Klebsiella from blood culture.  **1 patient diagnosed with GBS in both blood and cerebrospinal fluid  ***1 with meningitis (Neisseria meningitidis) | |

## Table 4: Infants with IBI misclassified by the CDAs Step-by-Step

| **Age category** | **Comorbidities** | **Hours from fever onset** | **Temperature recorded in ED** | **Unwell appearing** | **Neutrophil count (x 10*9/L)** | **C-Reactive protein (mg/L)** | **PCT (ng/ml)** | **Positive urinalysis** | **Organism (Bacteremia)** |
| --- | --- | --- | --- | --- | --- | --- | --- | --- | --- |
| **29 – 90 days** | No | <6hrs | 36.6 | No | 8.9 | 2 | 0.09 | No | Staphylococcus aureus |
